# Supplementary material for: Glomage: A Multimodal Platform for High‐Content Morphological and RNA Profiling of Glomeruli in Zebrafish and Mouse Models
Source: Adv Sci (Weinh). 2026 Apr 16;13(39):e23662. doi: 10.1002/advs.202523662 (PMC13326040; doi:10.1002/advs.202523662)
Supplement: Supplementary file 1 — Supporting File: advs75161‐sup‐0001‐SuppMat.pdf. [file ADVS-13-e23662-s001.pdf]

Supplemental Material for:

Glomage: A multimodal platform for high-content morphological and RNA profiling of glomeruli in zebrafish and mouse models

Maximilian Schindler[1], Tim Lange[1], Sophia-Marie Bach[1], Soeren S. Lienkamp[2,3,4], Nicole Endlich[1*]

[1] Department of Anatomy and Cell Biology, University Medicine Greifswald, Greifswald, Germany.

[2] Institute of Anatomy, University of Zurich, Zurich, Switzerland.

[3] Department of Health Sciences and Technology, ETH Zurich, Zurich, Switzerland.

[4] Zurich Kidney Center, University of Zurich, Zurich, Switzerland.

*Corresponding author: nicole.endlich@uni-greifswald.de

Table of contents

Supplemental Figures

1. Fig. S1
2. Fig. S2
3. Fig. S3
4. Fig. S4
5. Fig. S5
6. Fig. S6
7. Fig. S7

Supplemental Tables

1. Table S1
2. Table S2

Supplemental Movie Captions

1. Movie S1
2. Movie S2
3. Movie S3
4. Movie S4
5. Movie S5
6. Movie S6

Supplemental Figures

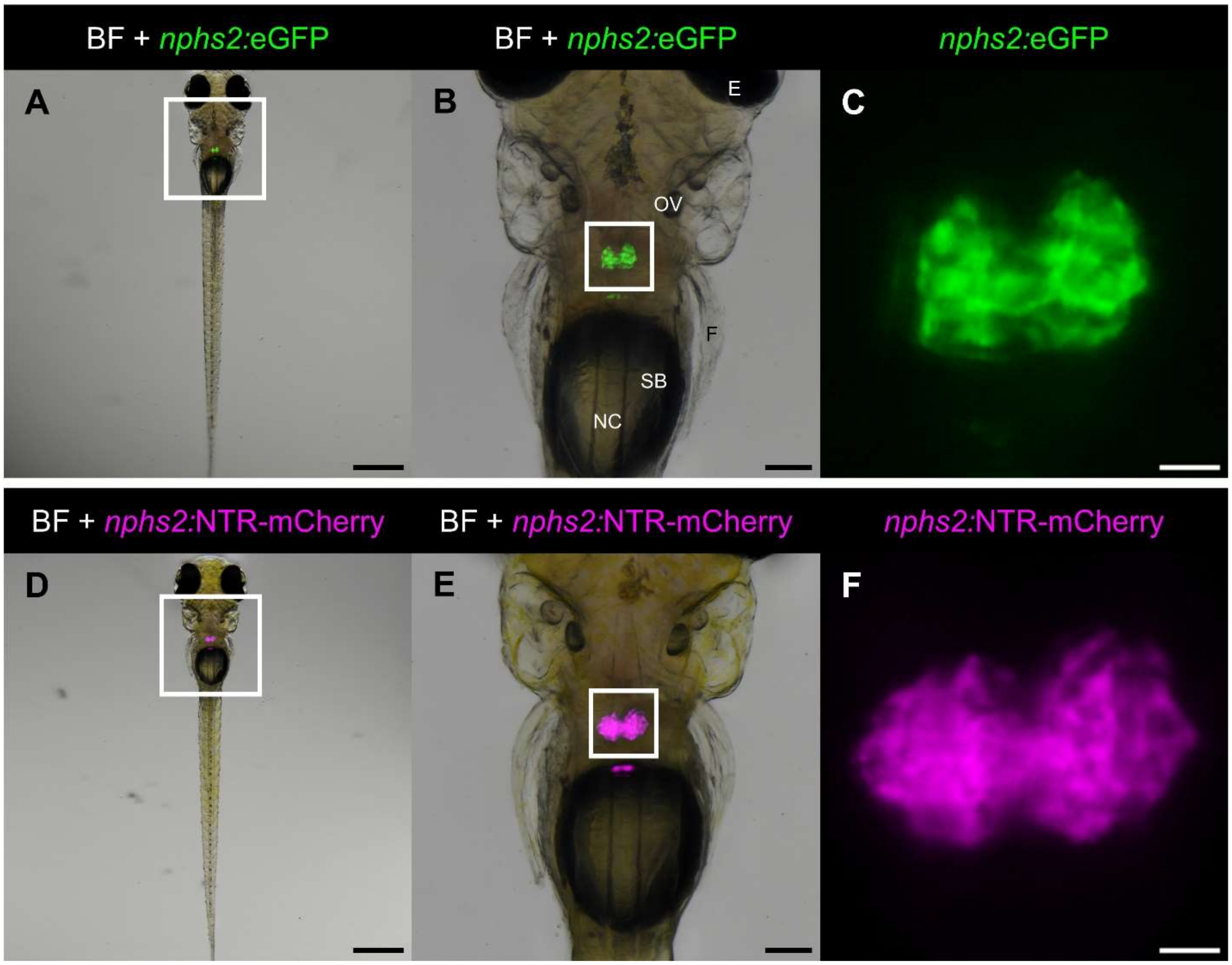

Figure S1: Overview of the transgenic zebrafish lines and their expression patterns. Larvae of the *Podo:GFP* line express eGFP exclusively in podocytes, the glomerulus is located cranial to the swim bladder and exhibits the typical peanut-like shape at 5 dpf (A–C). The *Cherry* strain shows the same expression pattern, with the fluorophore mCherry expressed in podocytes. In addition, this strain expresses *E. coli* nitroreductase (NTR) specifically in podocytes (D–F). NTR renders this strain sensitive to podocyte injury upon treatment with nifurpirinol (NFP). BF: Brightfield, E: Eye; OV: Otic vesicle; F: Fin; SB: Swim bladder; NC: Notochord. Scale bars: 500 µm (A, D); 100 µm (B, E); 20 µm (C, F).

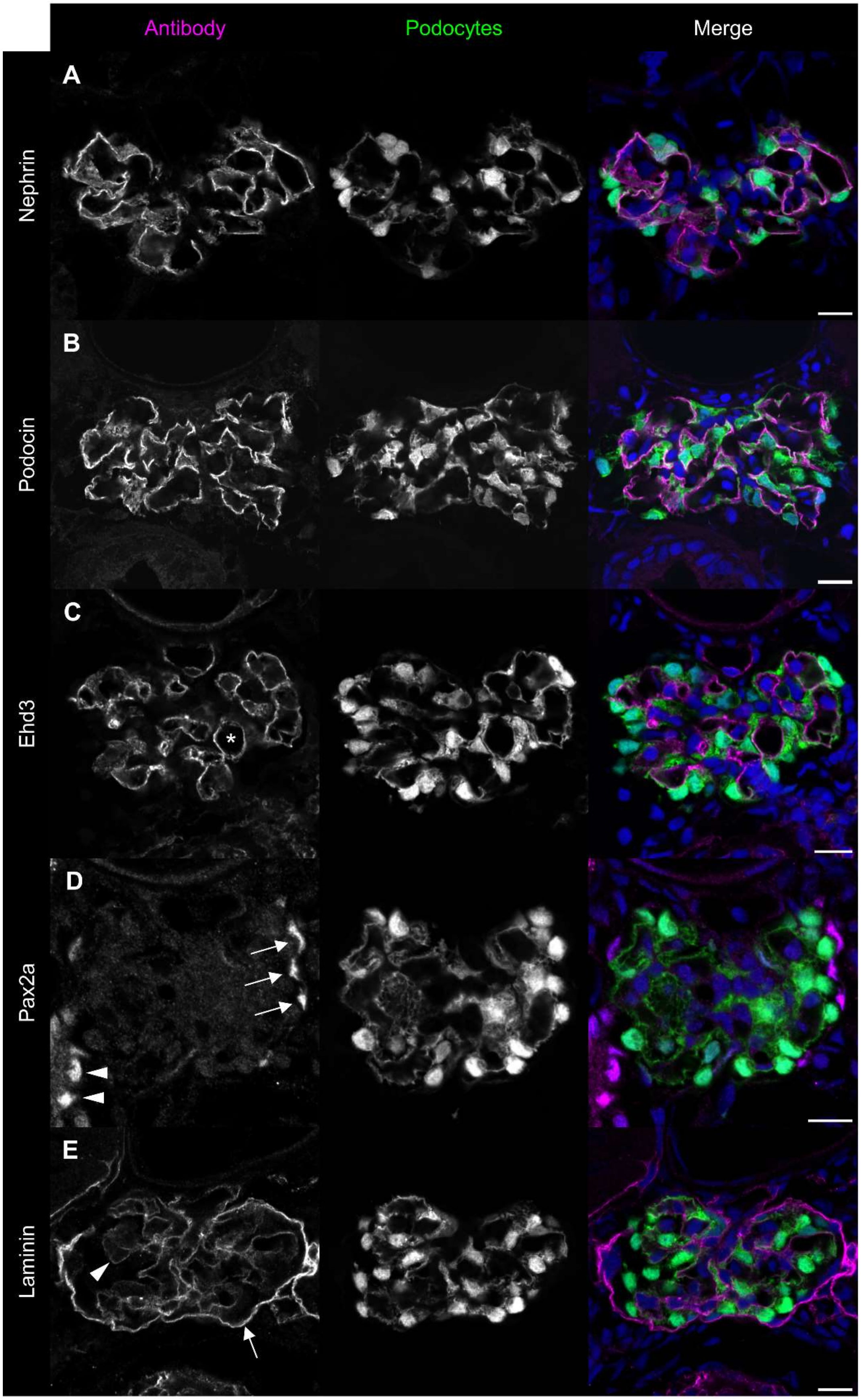

Antibody
Podocytes
Merge
A
Nephrin
B
Podocin
C
Ehd3
*
D
Pax2a
E
Laminin

Figure S2: Glomerular cryosections of *Podo:GFP* larvae stained with various antibodies. Nephrin and Podocin label the slit diaphragm of the glomerular filtration barrier. Ehd3 marks fenestrated glomerular endothelial cells revealing cross sections through glomerular capillaries (asterisk). Pax2 labels parietal epithelial cells (arrows) and proximal tubule cells (arrowheads). The Laminin antibody highlights basement membranes, including the parietal (arrow) and glomerular (arrowhead) basement membranes. Scale bars: 10 µm.

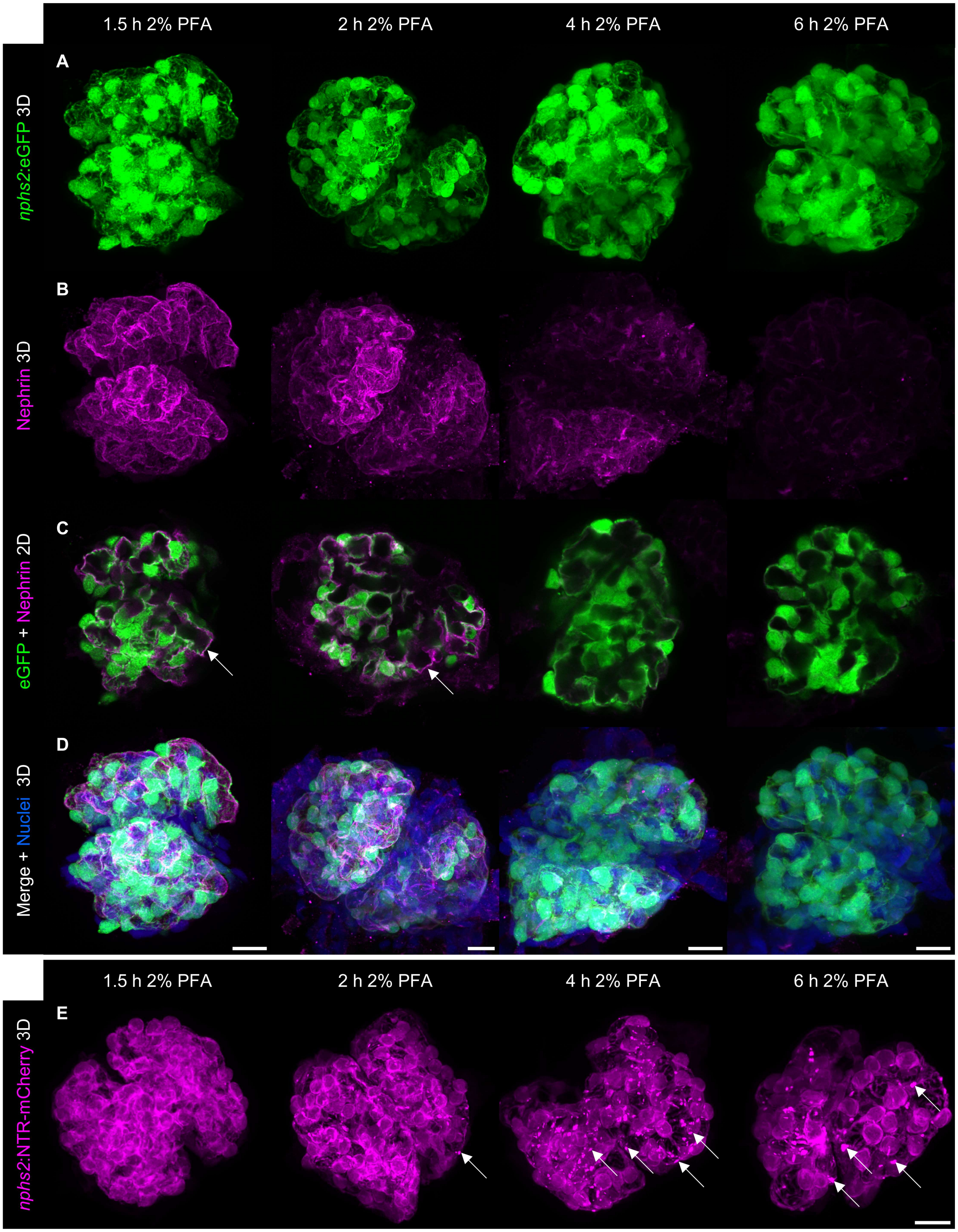

1.5 h 2% PFA
2 h 2% PFA
4 h 2% PFA
6 h 2% PFA
A
nphs2:eGFP 3D
B
Nephrin 3D
C
eGFP + Nephrin 2D
D
Merge + Nuclei 3D
1.5 h 2% PFA
2 h 2% PFA
4 h 2% PFA
6 h 2% PFA
E
nphs2:NTR-mCherry 3D

Fig. S3: Effect of PFA fixation time in the *Glomage* staining protocol. *Podo:GFP* larvae were fixed in 2% PFA for 1.5, 2, 4, and 6 hours. (A) Prolonged fixation had no detectable effect on the endogenous eGFP signal. (B) Immunostaining for the slit diaphragm protein nephrin showed strong and three-dimensional signal intensity after 1.5 hours of fixation, which progressively decreased with longer fixation times and was completely lost after 6 hours. (C) Single optical sections of z-stacks revealed the characteristic linear nephrin pattern along the glomerular filtration barrier in samples fixed for 1.5 and 2 hours (arrows), whereas the signal was absent after 4 and 6 hours of fixation. (D) Maximum intensity projections indicate that 1.5 hours fixation yields optimal three-dimensional staining results in the *Glomage* protocol. Larvae of the *Cherry* strain were processed under identical conditions. Maximum intensity projections of whole glomeruli show that prolonged fixation markedly affects the endogenous mCherry signal, with clustering becoming apparent after 2 hours of fixation (arrows). Glomeruli fixed for 4 and 6 hours exhibited pronounced mCherry clustering, resulting in highly inhomogeneous fluorescence signals (arrows, E). PFA: paraformaldehyde; h: hours. Scale bars: 10 µm.

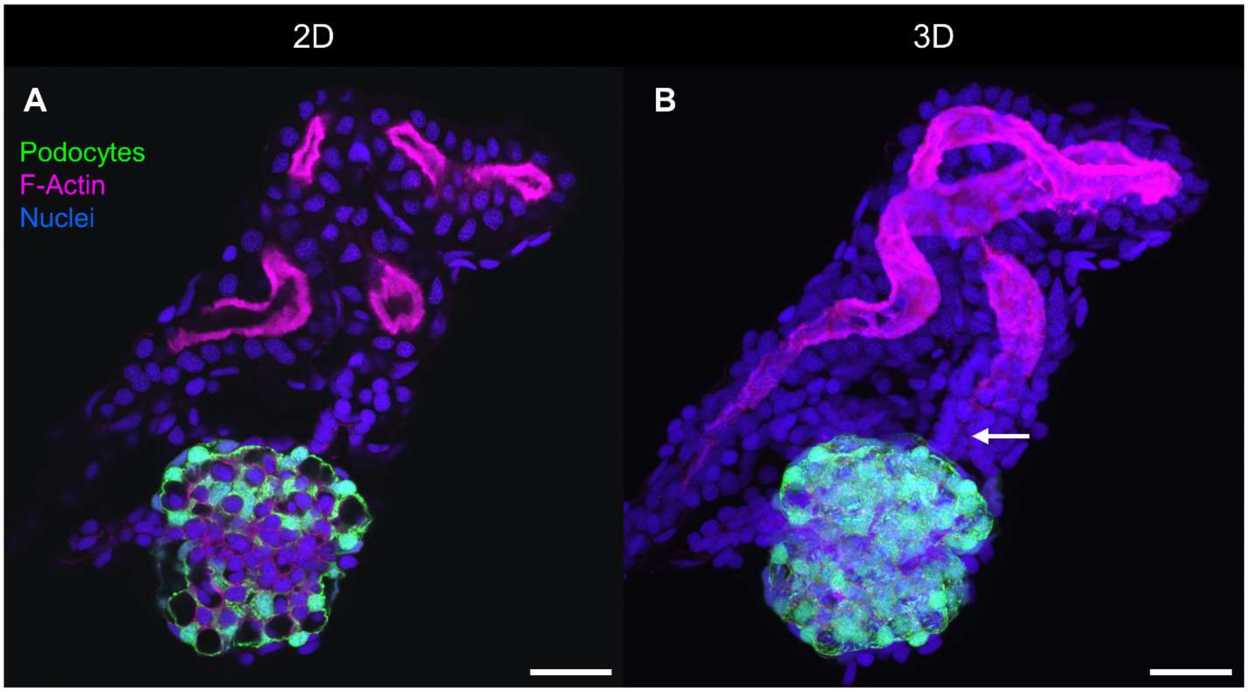

Figure S4: Two- and three-dimensional visualization of a glomerulus and tubule co-isolation. Podocytes are shown in green (*Podo:GFP*), while F-actin, labeled with Phalloidin, marks the brush border of proximal tubule cells (magenta). A single optical section shows different angles of a cut through the convoluted tubule (A). The MIP displays the entire convoluted tubule with its complete brush border (asterisk). The neck segment, which connects the tubule to the glomerulus, lacks a brush border (arrow) (B). Scale bars: 20 µm.

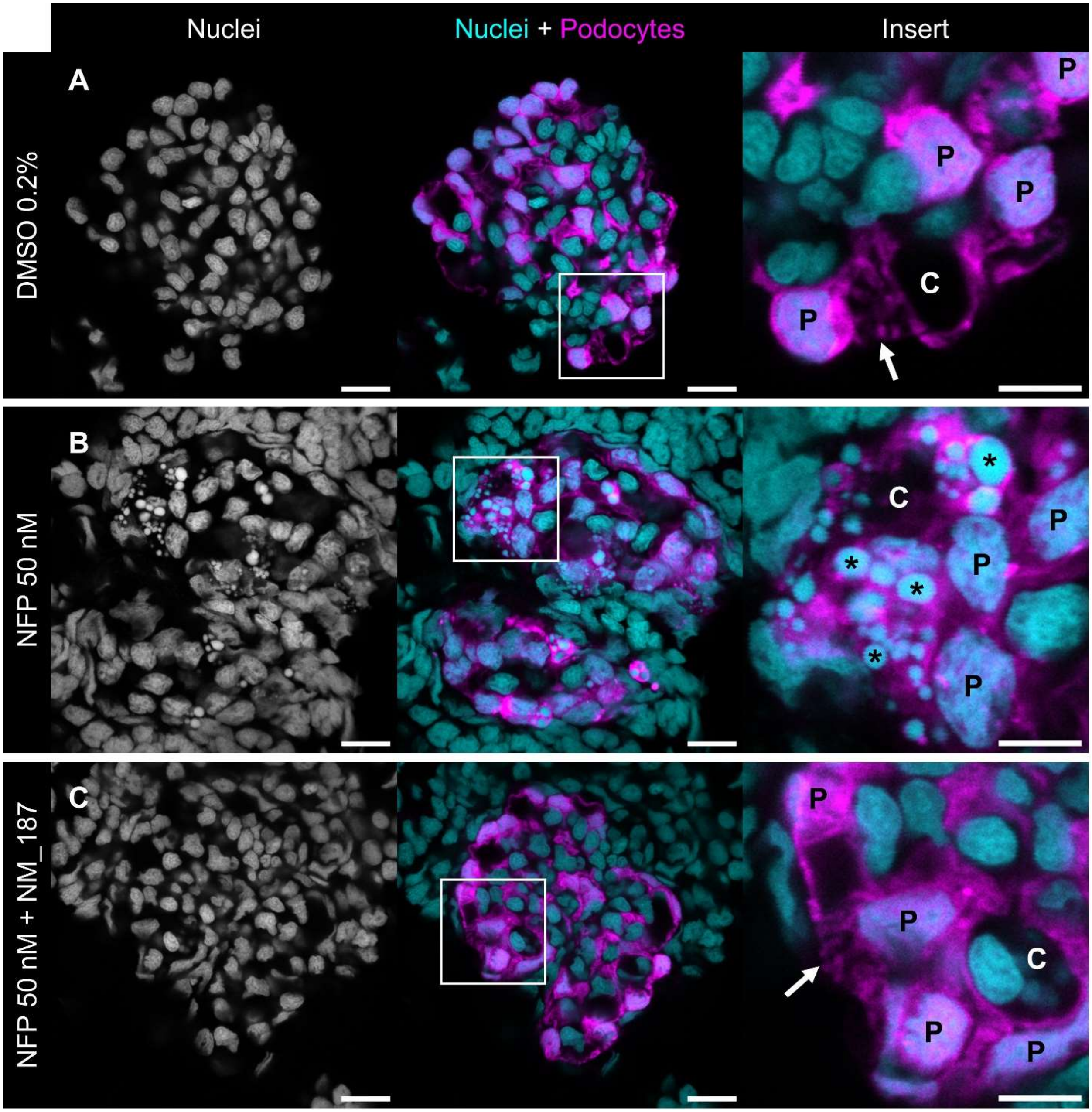

Figure S5: Single slices of whole glomeruli from *Cherry* larvae treated with DMSO, NFP, and NFP + NM_187. In the healthy glomerulus (DMSO), nuclei (cyan) appear normal, and primary processes of podocytes (magenta) are visible (arrow) (A). Larvae treated with NFP display fragmented podocyte nuclei (asterisks) 24 hours after injury induction (B). Nuclei of larvae treated with NFP + NM_187 appear healthy again, and primary processes are visible (C). C: Capillary; P: Podocyte. Scale bars in whole glomeruli images: 10 µm, Scale bars in inserts: 5 µm.

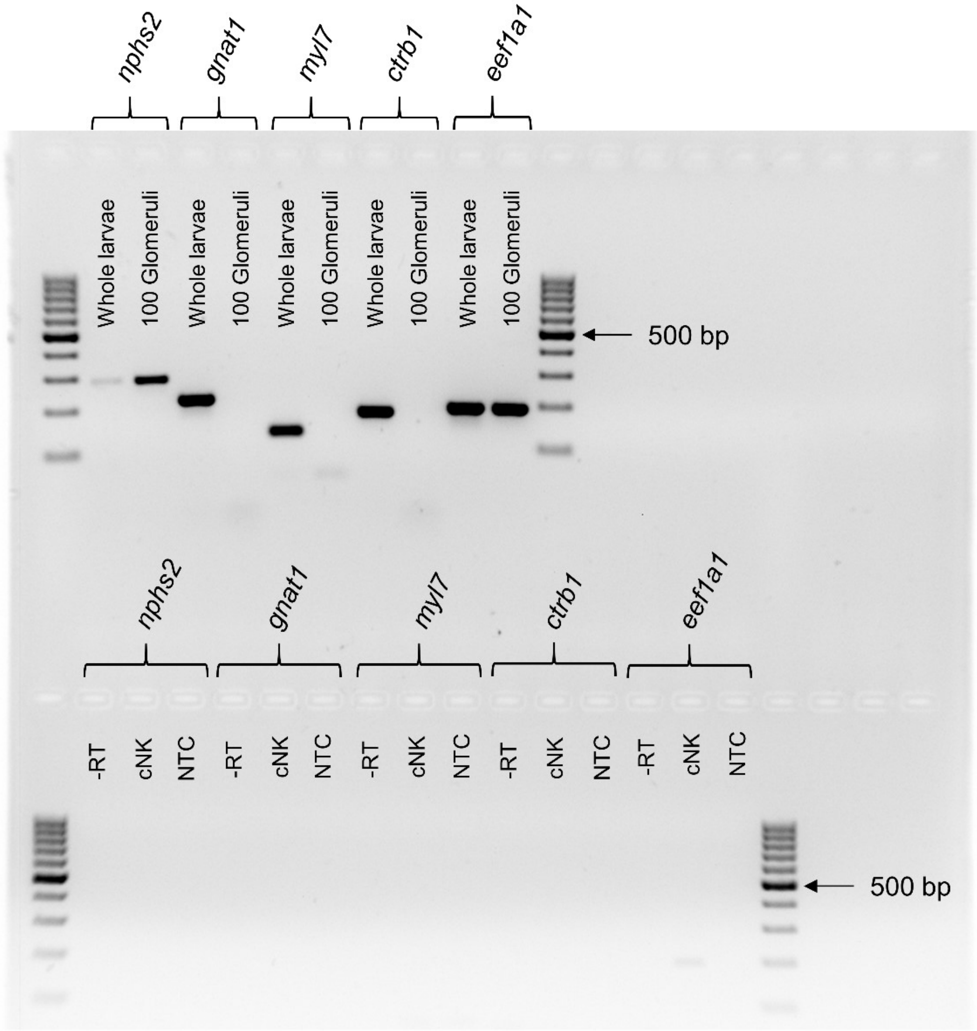

Figure S6: Corresponding uncropped RT–PCR gel image. The image shows the full agarose gel (2%) for the RT-PCR presented in Figure 5. The top row indicates the primer sets used and compares input from ten whole larvae and 100 isolated glomeruli. For all primer pairs, three controls were included: -RT (no reverse transcriptase during cDNA synthesis), cNK (no RNA during cDNA synthesis), and NTC (no-template control; water as PCR template).

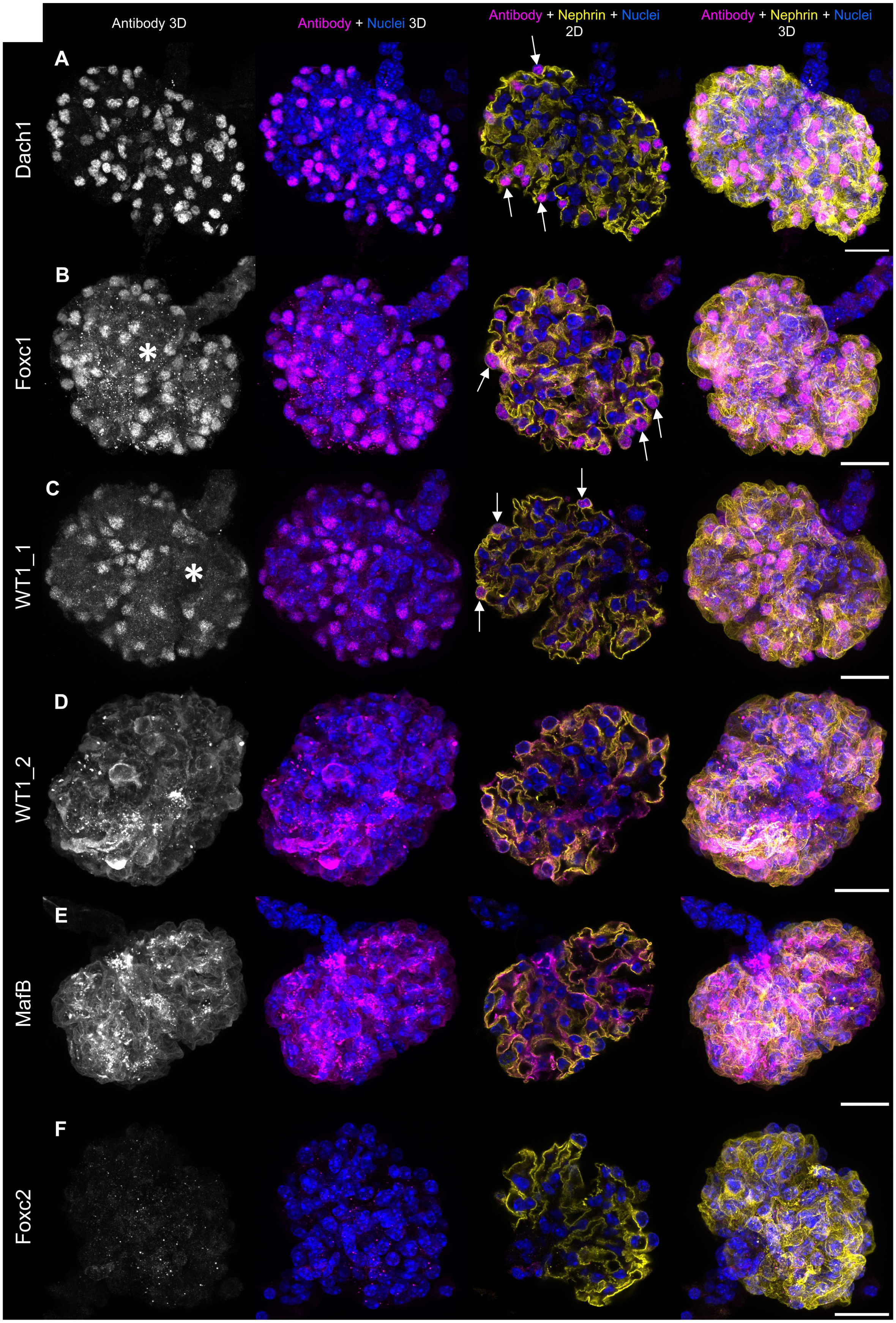

Antibody 3D
Antibody + Nuclei 3D
Antibody + Nephrin + Nuclei 2D
Antibody + Nephrin + Nuclei 3D
A
Dach1
B
Foxc1
C
WT1_1
D
WT1_2
E
MafB
F
Foxc2

Figure S7. Validation of antibodies for specific detection of podocyte nuclei in mouse glomeruli within the *Glomage* workflow. Immunofluorescence staining of isolated whole mouse glomeruli was performed using antibodies against selected nuclear podocyte markers. The Dach1 antibody exhibits a strong and highly specific nuclear signal with minimal background fluorescence (A). An antibody against Foxc1 also labels podocyte nuclei but displays moderate background signal compared to Dach1 (B). One of the tested WT1 antibodies (WT1_1) shows nuclear staining accompanied by substantial background fluorescence (C), requiring high laser intensity for signal detection even at high antibody concentration (1:100). In contrast, a second WT1 antibody (WT1_2) and an antibody against MafB do not produce specific nuclear staining in podocytes but instead show predominantly cytosolic signals in glomerular cells (D, E). An antibody against Foxc2 does not yield detectable signal (F). Asterisks indicate intraglomerular background in (B) and (C), white arrows highlight specific nuclear signals in single optical sections (2D) in (A–C). Scale bars: 20 µm.

Supplemental Tables

Table S1: List of primary antibodies used for immunofluorescence.

Zebrafish				
**Antigen**	**Provider**	**Cat. No.**	**Concentration**	**Performance**
Nephrin	Innovagen	NA	1:2000	+
Podocin	Sigma	PO372	1:7500	+
Pax2	Abcam	ab38738	1:2000	+
Ehd3	Sigma	HPA049986	1:200	+
Laminin	Sigma	L9393	1:100	+
**Mouse**				
**Antigen**	**Provider**	**Cat. No.**	**Concentration**	**Performance**
Nephrin	Progen	GP-N2	1:1000	+
Dach1	Sigma	HPA012672	1:500	+
Foxc1	Sigma	HPA040670	1:100	+/-
WT1_1	Santa Cruz	sc-192	1:100	+/-
WT1_2	Invitrogen	6FH2	1:100	-
MafB	Santa Cruz	sc-376387	1:100	-
Foxc2	Sigma	HPA056302	1:100	-

Table S2: Primers used for RT-PCR and RT-qPCR.

Gene	Tissue	Direction	Sequence	Amplicon
eef1a1l1	Housekeeping	Forward 5′–3′	AAGGAGGGTAATGCTAGCGG	190 kb
eef1a1l1	Housekeeping	Reverse 3′–5′	GGGCGAAGGTCACAACCATA	190 kb
nphs2	Podocytes	Forward 5′–3′	GGCCCTGGGCTGATGTTTTA	283 kb
nphs2	Podocytes	Reverse 3′–5′	GAGCAATGCGTTTCCTGTCC	283 kb
mCherry	Podocytes	Forward 5′–3′	GAAGAAGACCATGGGCTGGG	123 kb
mCherry	Podocytes	Reverse 3′–5′	TTGACCTCAGCGTCGTAGTG	123 kb
gnat1	Eye	Forward 5′–3′	TCGCTGCTCTGAGTGCATAC	218 kb
gnat1	Eye	Reverse 3′–5′	GAAGGTGTTGGGACCGTCAT	218 kb
myl7	Heart	Forward 5′–3′	CAGACCCAGAGGAAACCATCC	132 kb
myl7	Heart	Reverse 3′–5′	GGTCAACCTCTTCTGCTGTGA	132 kb
ctrb1	Pancreas	Forward 5′–3′	GGCTATTCAGACCATTGCCG	179 kb
ctrb1	Pancreas	Reverse 3′–5′	TCACACACTTCATGCCACCA	179 kb

Supplemental Movie Captions

Movie S1:

Optical sections through a larval zebrafish glomerulus. Podocytes are endogenously labeled with eGFP (green), and the slit diaphragm is detected with a nephrin antibody (magenta). Nuclei are shown in blue. The stack consists of 30 slices with 1 μm spacing and is recorded from ventral to dorsal at 2 frames per second. The larval glomerulus receives blood supply from the dorsal aorta, and the trench of this large vessel is visible in the last dorsal frames.

Movie S2:

Reconstruction of larval podocytes. This movie shows a rotation of all podocytes from a single glomerulus in a zebrafish larva. Podocytes are shown in green (eGFP) and major processes are visible.

Movie S3:

Reconstruction of the slit diaphragm. *Glomage* enables visualization of the entire slit diaphragm in glomeruli in 3D. The slit diaphragm protein nephrin (magenta) bridges the filtration slits between podocyte foot processes.

Movie S4:

Visualization of the proximal convoluted tubule. Co-isolated tubules attached to the glomerulus enable 3D visualization of the tubule structure and labeling of the brush border. Podocytes are shown in green (eGFP), F-actin is detected with 543-conjugated phalloidin (magenta), and nuclei are shown in blue. Cells of the neck segment, which connect the glomerulus to the tubules, display fewer apical microvilli compared to proximal tubule epithelial cells.

Movie S5:

Optical sections through a young (6 months) mouse glomerulus. A nephrin antibody labels the slit diaphragm (magenta), a Dach1 antibody detects all podocyte nuclei (yellow) and the nuclear countersatin was performed with DAPI (blue). The stack is composed of 17 slices with 1 μm spacing and runs at 2 frames per second.

Movie S6:

Three-dimensional reconstruction of a young mouse glomerulus. The reconstruction shows the three-dimensionality of the glomerulus, nephrin is depicted in yellow, Dach1 in magenta and nuclei in blue.
